# Changes in pediatric primary care contacts: has the RSV-immunization modified the age-related access?

**DOI:** 10.1186/s13052-026-02205-w

**Published:** 2026-02-02

**Authors:** Riccardo Boracchini, Elisa Barbieri, Benedetta Canova, Eugenio Baraldi, Carlo Giaquinto, Anna Cantarutti

**Affiliations:** 1 Department of Statistics and Quantitative Methods, Division of Biostatistics, Epidemiology and Public Health, University of Milan-Bicocca, Milan, Italy; 2 Department of Medicine and Surgery, University of Milan-Bicocca, Milan, Italy; 3 Department of Woman and Child Health, University of Padova, Padova, Italy; 4 Neonatal Intensive Care Unit, Department of Women’s and Children’s Health, Institute of Pediatric Research, University of Padua, Padua, Italy

**Keywords:** Primary care, Nirsevimab, Pediatrician contacts

## Abstract

**Background:**

Primary care is essential during children’s development, especially in the first years of life. Contacts with the Family Pediatricians (FP) always presented with seasonality, peaking in infants and toddlers. In Italy, nirsevimab was widely introduced in November 2024, potentially influencing the pattern of pediatric care utilization. This study aimed to evaluate temporal changes in FP contacts and the burden of bronchiolitis following nirsevimab introduction.

**Methods:**

A retrospective observational study has been conducted within Pedianet, an Italian primary-care network of FPs. Children residing in the Veneto region under five years of age and active between September 2022 and 2025 were included. All FP contacts were considered, identifying bronchiolitis-related visits and immunization ones. Age-specific incidence rates (IR) expressed in 100 person-months with 95% confidence intervals (CI) were calculated.

**Results:**

A total of 25,399 children (median follow-up: 20.99 months) were included. Overall, 71.68 (95%CI:71.46–71.91) contacts per 100 person-months were recorded, showing clear seasonality with decreasing incidence while increasing age. After excluding immunization contacts, the difference in IRs between children in their first and second year of life was smaller in 2024/2025 than in 2023/2024, particularly in November (1-year:88.96 [95%CI:85.78–92.14] and 2-year:84.68 [95%CI:81.40–87.95] compared with 119.3 [95%CI:115.47–123.12] and 94.19 [95%CI:90.72–97.66]). Bronchiolitis burden among infants < 1 year declined from 6.58 (95%CI:5.70–7.47) to 1.24 (95%CI:0.87–1.62) contacts per 100 person-months in January 2024 and 2025, respectively, with a delayed seasonal peak in February 2025 (IR = 2.00, 95%CI:1.50–2.50).

**Conclusions:**

Following nirsevimab introduction, the burden of bronchiolitis markedly decreased, and differences in early-life contact rates narrowed. These findings support the need for adaptive pediatric care planning and continuous surveillance of respiratory disease trends accounting for preventive strategies implemented at the community level.

**Supplementary information:**

The online version contains supplementary material available at 10.1186/s13052-026-02205-w.

## Introduction

Primary care, especially in pediatrics, plays a crucial role in children’s health by providing preventive services, monitoring growth and neurodevelopment, and managing both acute and chronic diseases throughout the first years of life [1, 2]. The use of primary care varies significantly by age, with a higher number of consultations typically occurring during infancy and early childhood due to infections, vaccinations, and scheduled visits [3]. In Italy, well-child visits scheduled by the Italian National Health System (NHS) are delivered by family pediatricians (FPs) to monitor growth, development, and overall health, with ten visits occurring mainly in the first two years of life [4].

As children grow, the number and nature of primary-care visits evolve. Infants and toddlers mostly present with infectious diseases and require regular check-ups and follow-up visits, whereas school-aged children often access ambulatory services for injuries, certificates, or behavioral concerns. Seasonal patterns also influence the demand for primary care. During the epidemiological peak, the circulation of respiratory viruses (e.g., respiratory syncytial virus (RSV), influenza virus) contributes to the fluctuation in visit burden, especially for respiratory infections among infants and toddlers [5].

In recent years, shifts in viral epidemiology following COVID-19 and the adoption of new immunization strategies have altered patterns of pediatric healthcare utilization. In particular, the introduction of nirsevimab immunization for all newborns in the 2024–2025 season in Italy changed the epidemiology of lower RTI [6]. Understanding these changes is crucial to help FPs reorganize care and optimize resources.

Nirsevimab has been administered nationwide in Italy since November 2024 to all newborns up to March 2025. However, differences in implementation emerged based on regional policies and healthcare decisions. In the Veneto region, nirsevimab was also offered to children born between January and October 2024 facing their first RSV season. Regional coverage was high, reaching 70–80% of children eligible for immunization, which may have contributed to reduced viral circulation and fewer LRTI cases in younger children.

This study aimed to explore patterns of pediatric primary-care contacts, with particular focus on whether the age distribution of children presenting to FPs changed after the introduction of nirsevimab in Italy.

## Methods

### Data source

Children were enrolled from Pedianet (http://www.pedianet.it), a network of over 250 FPs using a standardized primary care database (Junior Bit®). The database anonymously collects demographic, clinical, and prescription data from children enrolled by participating FPs whose parents/guardians have provided informed consent, representing approximately 4% of the annual national paediatric population.

### Study design and population

We conducted a retrospective observational study to assess changes in pediatric primary care encounters over time and across age classes. Children under five years residing in the Veneto region, who adhered to well-child visits and were active between 2022/2023 and 2024/2025 epidemiological seasons (each epidemiological season is considered from September 23, 20XX to September 22, 20XX+1), were included. The Veneto Pedianet cohort represents about 15% of the Veneto annual pediatric population. Children in care of FPs, who were already involved in prospective observational studies were excluded.

### Outcome definition

The primary outcome of interest was defined as all primary-care contacts with FPs. Immunization-related contacts (influenza vaccination and nirsevimab, the only immunizations administered in the FP ambulatory setting) were also excluded from the overall contact count. In addition, a specific outcome related to bronchiolitis diagnoses was considered, identified using a machine learning algorithm (Supplementary Materials, Appendix I).

## Statistical methods

Demographic and social characteristics of children, antibiotic prescriptions and the outcome of interests were described with number and frequencies and with median and interquartile ranges, as appropriate. Incident rates (IR), with corresponding 95% confidence intervals (95% CI), of primary-care contacts with and without immunization as well as contacts for bronchiolitis diagnoses were reported per 100 person-months. Each subject was followed since birth or the enrolment date until the end of assistance or until April 30th 2025, whichever came first. IR were stratified according to children’s age from 0 to 5 years. We followed the STrengthening the Reporting of OBservational studies in Epidemiology (STROBE) guidelines. All statistical analyses were performed using SAS software, version 9.4 (SAS Institute, 17 Inc., Cary, NC, USA).

## Results

A total of 25,399 children were included in the cohort (51.77% males), with a median age at study entry of 11.27 months (IQR: 0.26–35.29). Half (50.52%) entered during their first year of life. Median follow-up was 20.99 months (IQR:10.78–32.56). During follow-up, 7.28 (95%CI: 7.21–7.36) antibiotic prescriptions and 71.68 (95%CI: 71.46–71.91) contacts per 100 person-months were recorded. Overall, 50.49% received at least one antibiotic, and 52.33% accessed primary care more than 10 times (Table S1).

**Fig. 1:**
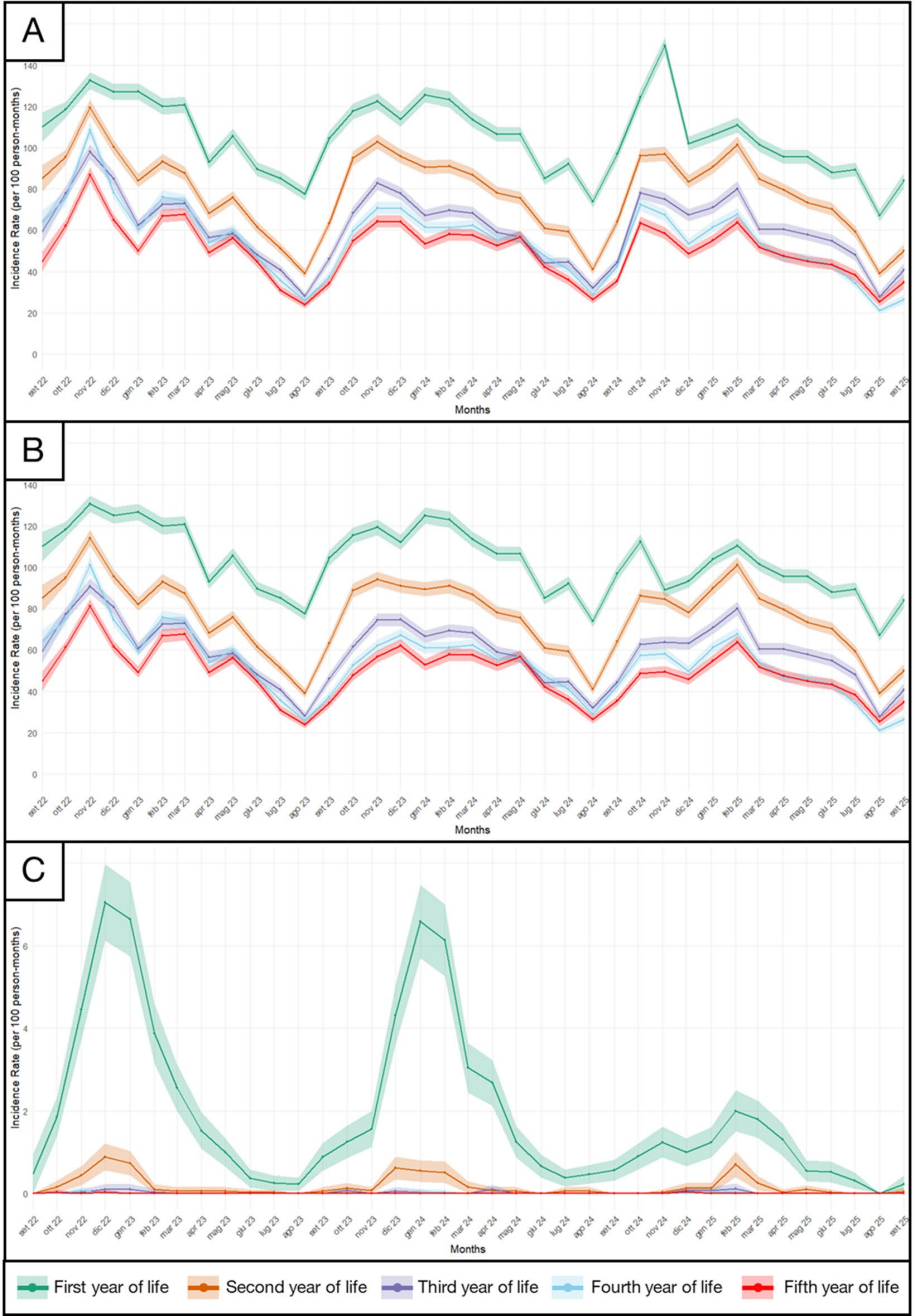
Panel A displays the contacts with the FP’s incidence rate; Panel B displays the contacts with the FPs’ incidence rates without considering immunizations; Panel C displays the burden of bronchiolitis visits incidence rates. All the incidence rates have been expressed in 100 person-months

Contacts incidence rates showed clear seasonal patterns and decreased with increasing age (Fig. 1 - Panel A). After excluding immunization contacts, the difference between children in their first and second year of life was smaller in 2024/2025 than in 2023/2024. In November 2024/2025, rates were 88.96 (95% CI: 85.78–92.14) contacts per 100 person-months in the first year of life and 84.68 (95% CI: 81.40–87.95) in the second year. In contrast, in November 2023/2024 the corresponding rates were 119.3 (95% CI: 115.47–123.12) and 94.19 (95% CI: 90.72–97.66), respectively (Fig. 1 - Panel B).

Bronchiolitis-related contacts declined sharply in 2024/2025 among infants under one year from 6.58 (95%CI: 5.70–7.47) visits per 100 person-months in January 2024 to 1.24 (95%CI: 0.87–1.62) visits in January 2025, with a delayed seasonal peak of 2.00 (95%CI: 1.50–2.50) in February 2025. Conversely, children in their second year maintained a similar seasonal peak in 2024/2025 compared to the previous epidemiological season (0.61, 95%CI: 0.34–0.89 in December 2023 vs. 0.70, 95%CI: 0.40–1.01 in February 2025) with a gradual decline in the overall season incidence over the seasons (Fig. 1 – Panel C).

## Discussion

In this primary care-based observational study of over 25,000 children in the Veneto region of Italy, we found that following the introduction of nirsevimab immunoprophylaxis, the burden of bronchiolitis markedly decreased among infants ( < 1 year), while the overall seasonal pattern of FPs’ contacts persisted, with a reduced incidence in early life.

These results are consistent with emerging evidence on the effectiveness of nirsevimab in preventing RSV-related illness in children, with increasing attention to its role in primary care. Beyond the pivotal HARMONIE [7] and MELODIE [8] trials showing 80% efficacy in controlled settings, real-world studies have confirmed broader benefits. Nirsevimab has been shown to prevent severe RSV-related infections requiring medical care [9], to reduce RSV infections and ambulatory visits in test-negative designs [10], and to provide protection across healthcare settings in the first real-world review of universal prophylaxis [11]. Our findings add to this body of evidence by documenting a shift in the burden of care at the primary care level, with fewer bronchiolitis visits in infants.

The reduced incidence of bronchiolitis among children under one year of age in the Veneto region likely reflected both the protection conferred by the nirsevimab campaign and a modification in community viral circulation. Emerging literature supports this assumption. A Spanish multicentre prospective study, where nirsevimab was introduced one year earlier than in Italy, showed a concomitant 20–30% decrease in RSV-associated bronchiolitis and a 10–20% increase in rhinovirus- and metapneumovirus-associated infection, suggesting viral pathogen substitution and shifts in the epidemiology and seasonality of respiratory infections [12].

This study has several strengths. First, the larger sample size in a real-world primary care setting allowed us to estimate the burden outside hospitals, providing a clearer picture that is generalizable to similar pediatric primary care settings and informative for healthcare providers. Second, a three-year follow-up period across RSV seasons enabled us to isolate changes that occurred specifically after the introduction of nirsevimab.

Our findings have concrete implications for the primary care management, planning, and pediatric resource allocation. FPs need to be informed and aware of possible shifts in visit burden due to the virus circulation modification and the possibility of visiting older children with a lower-risk profile. NHSs need to be prepared for new preventive education and follow-up strategies. Furthermore, age-specific surveillance systems are essential for determining the new seasonal pattern and understanding the long-term effects of nirsevimab immunization.

## Conclusion

In conclusion, the introduction of nirsevimab in the Veneto region has been associated with a substantial reduction in the burden of bronchiolitis among children under one year of age. As new preventive interventions become part of standard care, they have the potential to reshape pediatric primary care demand, highlighting the need for adaptive strategies.

## Electronic supplementary material

Below is the link to the electronic supplementary material.

Supplementary Material 1

## Data Availability

Data may be obtained from a third part and are not publicly available.
