# Supplementary material for: Changes in pediatric primary care contacts: has the RSV-immunization modified the age-related access?
Source: Ital J Pediatr. 2026 Feb 2;52:32. doi: 10.1186/s13052-026-02205-w (PMC12955257; doi:10.1186/s13052-026-02205-w)
Supplement: Supplementary file 1 — Supplementary Material 1 [file 13052_2026_2205_MOESM1_ESM.docx]

**Supplementary Materials**

Changes in pediatric primary care contacts: has the RSV-immunization modified the age-related access?
Authors

Riccardo Boracchini^1,2*^, Elisa Barbieri^3^, Benedetta Canova^1,2^, Eugenio Baraldi^4^, Carlo Giaquinto^3¥^, Anna Cantarutti^1¥^

^¥^ Contributed as co-last

*Correspondence: Riccardo Boracchini, [riccardo.boracchini@unimib.it](mailto:riccardo.boracchini@unimib.it), (+39 345 3242013)

Index

[**Appendix I** – Outcome definition 2](#_Toc216426374)

[**Table S1.** Sociodemographic and access to primary-care characteristics of children enrolled into the study expressed in N (%) or median (Q1-Q3). 4](#_Toc216426375)

[**References** 5](#_Toc216426376)

# Appendix I – Outcome definition

A typical text classification pipeline includes data pre-processing, feature extraction, and machine learning classifiers^1^. The approach used for creating the classification pipeline focused on supervised machine learning and the text was numerically represented using the bag-of-word approach. For the bronchiolitis classifier, a labelled training dataset was created by extracting a subset of records which were manually reviewed and annotated by a clinician with binary labels indicating the presence or absence of the disease. Records containing a bronchiolitis diagnosis or the ICD-9-CM code related to the disease were labelled as positive; all others were labelled as negative. Given the higher prevalence of non-bronchiolitis cases, the dataset was balanced using undersampling techniques to ensure equal representation of both classes. The obtained training dataset was a balanced dataset comprising 200 documents labeled as bronchiolitis and 200 labeled as non-bronchiolitis. Preprocessing involved removing Italian stop words, tokenization, and extracting unigrams, applying a minimum document frequency threshold of 2 to reduce vocabulary size. A dual-branch pipeline was implemented, with one branch processing the free-text diagnosis field (primary, secondary, and tertiary diagnoses) and the other handling the structured ICD-9-CM codes (primary, secondary, and tertiary diagnostic codes). Both branches employed a Bag-of-Words representation via CountVectorizer, and their outputs were merged using a ColumnTransformer (i.e., the document-term-matrices were computed separately and then horizontally stacked). The extracted features were then passed to a logistic regression classifier with a regularization parameter C equal to 0.001.

The hyperparameters were determined through a grid search applied on the previously described dataset. These optimized parameters were subsequently used in the described parallel pipeline.

Model robustness was assessed through 5-fold stratified cross-validation. In each fold, predictions were generated and stored, enabling the assessment of aggregate performance. The model's performance was evaluated with various classification metrics, such as accuracy, precision, F1-score, sensitivity, and specificity. Afterward, the trained classifier was deployed on an separate, unlabeled dataset, and its predictions manually reviewed by a clinician to evaluate the model's generalizability. Whenever problems were identified, additional labelled records were incorporated into the training set, and the model was retrained. This iterative refinement process was repeated as necessary to improve both the training data and the classifier’s overall performance. The model was evaluated on a new subset, randomly extracted, whose labels were manually reviewed by a clinician and that was not used during training. The final model achieved optimal performance, with all the metrics approximately equal to 1.

Operative characteristics of the selected algorithm for bronchiolitis classification into the Pedianet database.

|  | **Precision** | **F1-score** | **Sensitivity** | **Specificity** | **Accuracy** |
| --- | --- | --- | --- | --- | --- |
| **Bronchiolitis** | 1.00 | 1.00 | 1.00 | 1.00 | 1.00 |
| **Non-bronchiolitis** | 1.00 | 1.00 | 1.00 | 1.00 |  |

# Table S1. Sociodemographic and access to primary-care characteristics of children enrolled into the study expressed in N (%) or median (Q1-Q3).

|  | Veneto cohort |
| --- | --- |
|  | N = 25399 |
| Sex |  |
| Female | 12249 (48.23) |
| Male | 13150 (51.77) |
| Age in months at cohort entry, median (IQR) | 11.27 (0.26 - 35.29) |
| < 1 year | 12832 (50.52) |
| 1 year | 3359 (13.22) |
| 2 years | 3125 (12.30) |
| 3 years | 3099 (12.20) |
| 4 years | 2984 (11.75) |
| Months of FU time by age class, median (IQR) | 20.99 (10.78 - 32.56) |
| < 1 year | 21.06 (11.37 - 33.41) |
| 1 year | 35.71 (29.67 - 35.98) |
| 2 years | 28.59 (24.71 - 32.59) |
| 3 years | 16.76 (13.74 - 20.71) |
| 4 years | 6.51 (3.17 - 9.65) |
| Antibiotic assumpion during FU* | 7.28 (7.21 – 7.36) |
| No antibiotics | 12575 (49.51) |
| At least one antibiotic | 12824 (50.49) |
| Number of visits during FU* | 71.68 (771.46 – 71.91) |
| 0 - 5 | 7144 (28.13) |
| 6 – 10 | 4964 (19.54) |
| > 10 | 13291 (52.33) |
| *Incidence rates expressed in 100 person-months | |

# References

1. Revina A, Buza K, Meister VG. Designing Explainable Text Classification Pipelines: Insights from IT Ticket Complexity Prediction Case Study. In: 2021:293-332. doi:10.1007/978-3-030-64949-4_10
